# Distributions of Potential and Contact-Induced Charges in Conventional Organic Photovoltaics

**DOI:** 10.3390/ma13102411

**Published:** 2020-05-24

**Authors:** Kouki Akaike

**Affiliations:** National Institute of Advanced Industrial Science and Technology, Tsukuba 305-8565, Japan; kouki.akaike@aist.go.jp; Tel.: +81-029-849-1221

**Keywords:** donor/acceptor interface, electrode interface, energy-level alignment, dark charges, organic solar cells

## Abstract

The interfaces of dissimilar materials play central roles in photophysical events in organic photovoltaics (OPVs). Depth profiles of electrostatic potential and contact-induced charges determine the energy-level lineup of the frontier orbitals at electrode/organic and organic heterointerfaces. They are critical for the elementary processes in an OPV cell, such as generation and diffusion of free carriers. A simple electrostatic model describes the energetics in organic heterojunctions supported by an electrode, and experiments via photoelectron spectroscopy and the Kelvin probe method validate the potential distribution in the stacking direction of the device. A comparative study has clarified the significance of Fermi-level pinning and resulting electrostatic fields in determining the energy-level alignment. In this review, we discuss how parameters of device constituents affect the distributions of potential and the dark charges in conventional OPVs comprising metallophthalocyanine and C_60_ as donor and acceptor, respectively. The results of previous studies, together with additional numerical simulations, suggest that a number of the factors influence the depth profiles of the dark charge and potential, such as the work function of bottom materials, layer thickness, structural inhomogeneity at interfaces, top electrode, and stacking sequence.

## 1. Introduction

Organic photovoltaics (OPVs) have attractive potentials that ubiquitously harness solar energy. Benefits of OPVs, such as being lightweight, their flexibility, color tunability, and the low-cost production, have enabled steady progress in utilizing the devices for wearable electronics [1], self-powered buildings, and solar-powered automobiles with an option of the use of semitransparent solar cells for windows and skylights [2,3]. The development of new dyes and interface materials, and the controlling morphology of photoactive layers have contributed to improvements in photoelectric conversion and device stability [4]. Use of non-fullerene acceptors, in particular, boosts power conversion efficiency to over 17% [5] and even 18% for a single-junction OPV cell [6]. The performance of OPVs in state-of-the-art applications relies on the optoelectronic properties of their constituents [3,4,7,8]. Large absorption coefficients in the visible-light range and sufficient charge mobility are prerequisites for donor and acceptor. Formation of Frenkel excitons and transport of photogenerated charges are determined by these parameters. Key electronic processes in OPVs, such as free-charge generation/recombination and extraction, on the other hand, occur at the interfaces of molecular semiconductors and electrodes. Energy levels play decisive roles in the elementary events: energy offsets of the frontier orbitals at the donor/acceptor interface are required to form charge-transfer (CT) states that are precursors of free charges. As for charge extraction, built-in potential at electrode/organic interfaces can assist free charges swept toward anode and cathode [9,10]. Considering these aspects, experimental demonstration and mechanistic modeling of energy-level alignment are essential for the rational design based on respective device constituents. Photoelectron spectroscopy and the Kelvin probe method (KP) have been used to directly measure the energy-level shifts at the interface of organic semiconductors [11,12,13,14,15,16,17]. A number of ultrahigh vacuum (UHV)-based studies via ultraviolet photoelectron spectroscopy (UPS) and KP have revealed energy shifts over the nominal thickness of several tens of nanometers from electrode/organic and organic heterointerfaces. Origins of the energy shift were typically interpreted as a consequence of the ground-state charge transfer at the interfaces. The formation of interface dipole may account for the observed energy shift [12,18]. However, the magnitude of the shift near the interfaces largely depends on the position of the Fermi level (*E*_F_) of a bottom layer [15,19,20]. Moreover, non-monotonic energy shifts have been reported for donor/acceptor interfaces [13,16,21]. These observations were not explained by solely assuming the formation of the dipole layer. An electrostatic model reported by Oehzelt et al. [22] comprehensively substantiated these observations by quantifying profiles of the contact-induced charge density that is also given in these model simulations.

Complementary studies employing photoemission spectroscopy and these simulations based on the model have clarified the decisive roles of the Fermi-level pinning and induced electrostatic fields in determining energetic landscape at organic semiconductor interfaces [21,23,24]. Based on these findings, we will discuss how parameters of the device, such as layer thickness, etc., influence the behavior of Fermi-level pinning and then potential/contact-induced charge distributions at the electrode/organic and organic donor/acceptor interfaces. Analysis of the charge density profile along the stacking direction explains the modification of potential distributions through presence of structural disorder and top electrodes. The electrostatic simulations detail the mechanism of the changes in the distributions upon the contact of heteromolecules. We chose metallophthalocyanine and C_60_ as a prototypical donor and acceptor used in conventional OPVs, respectively. One of benefits of the use of these molecules in basic research is that the electronic structure of the materials is already known [25,26,27,28]. Moreover, comprehensive understanding on the distributions of potential and charge density in conventional OPVs will be helpful to differentiate between recent molecules that enable barrierless charge density separation [29] from prototypical organic semiconductors.

## 2. Charged Molecules at Electrode/Organic Interfaces in the Ground State

Organic semiconductors are often used without intentional doping. The intrinsic charge density (*n*_i_) of an organic film was calculated from the energy gap, spin degeneracy, and dispersion of Gaussian functions, modeling the highest occupied molecular orbital (HOMO) and lowest unoccupied molecular orbital (LUMO) of a molecule [30]. Assuming Boltzmann distribution and equal density for thermally excited holes and electrons, *n*_i_ for metal-free phthalocyanine (H_2_Pc) was estimated to be 1.0 × 10^8^ cm^−3^. Such a low value was actually demonstrated for a H_2_Pc crystal (8.86 × 10^6^ cm^−3^) [31]. The low value of *n*_i_ is simply due to the large energy gap of the molecule. In practical devices such as an OPV cell, a molecular semiconducting film is sandwiched between bottom and top electrodes. The surfaces of the two electrodes are in general modified with organic or inorganic material that tune the work function in the range of 2.5–7 eV and make electrode contacts ohmic. Analysis of photoelectron spectra demonstrated the presence of charged species right at the electrode interface [32,33,34,35,36]. Molecular cations are present at the interface with high-work function substrates, such as MoO_3_-covered metal [35,36], while anions appear at the electron-injection/extraction interface [32].

A numerical model that assumes the Fermi-Dirac occupation [22] rationalized the formation of the charged species near the electrode interfaces. In brief, the model simulates depth-resolved distributions of electrostatic potential and charge density in ground states. The model assumes that the bottom electrode is an electron reservoir with a constant chemical potential, namely, *E*_F_; electron occupation obeys the Fermi-Dirac statistics under thermodynamic equilibrium through the heterostructure. Therefore, electron exchange can freely occur between the organic layer and the bottom electrode. The resulting charges in the organic layers, ideally formed in the Frank-van der Merwe mode, are calculated using:

(1) $$\rho \left(z\right)=e\cdot n\cdot \left\{\int_{-\infty }^{\infty }f_{h}\left(E\right)\cdot D_{H}\left[E+eV\left(z\right)\right]dE-\int_{-\infty }^{\infty }f_{e}\left(E\right)\cdot D_{L}\left[E+eV\left(z\right)\right]dE\right\}$$

where *e* is the elementary charge, *n* is the molecular number density per area layer, and *f*_h_ and *f*_e_ are the Fermi–Dirac functions for holes and electrons, respectively. *D*_H_ and *D*_L_ are the density of states (DOS) shapes for the HOMO and LUMO, respectively. *V*(*z*) is the potential at the distance z from the substrate surface. The one-dimensional Poisson equation gives the potential of respective organic layers.

(2) $$\nabla \left(\varepsilon \left(z\right)\nabla V\left(z\right)\right)=-\frac{\rho \left(z\right)}{\varepsilon_{0}}$$

where *ε*_r_(*z*) is the dielectric constant at the distance *z* and *ε*_0_ is the permittivity in a vacuum. The obtained potential is used to again calculate charges by solving Equation (1), and then a new *V*(*z*) is calculated from Equation (2). This self-consistent calculation can simulate depth-resolved charge density and potential distributions. Numerical simulations were able to reproduce well the observed energy shifts by photoemission spectroscopy for electrode/organic [32] and organic heterointerfaces [21,23,24]. All calculations were carried out using Mathematica software (12.0.0.0, Wolfram Research, Champaign, IL, USA).

**Figure 1:**
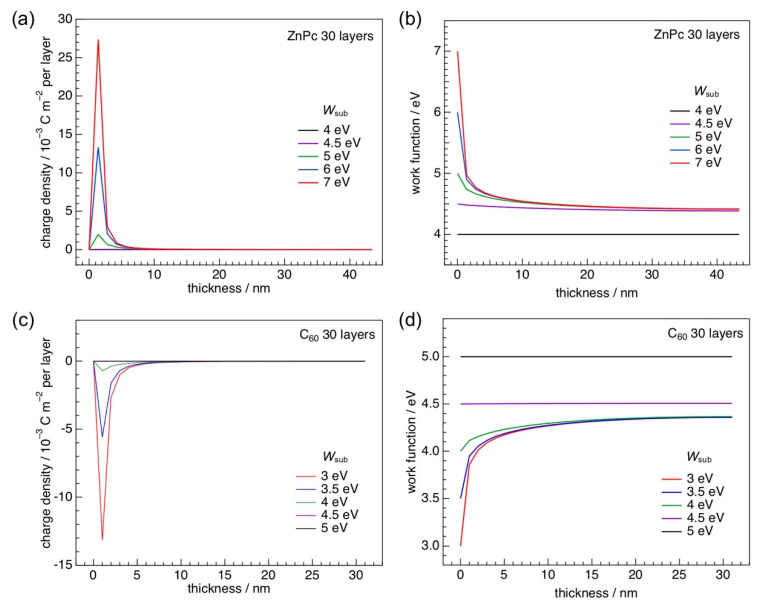
(**a**) Evolution of charge-density distributions in a ZnPc layer on a substrate with work functions of 4–6 eV as a function of film thickness. (**b**) Corresponding potential distributions are shown. ZnPc molecules are assumed to adopt edge-on orientation that is typically observed for films prepared on practical electrodes for organic photovoltaics (OPVs). (**c**) Evolution of the work function in a C_60_ layer on a substrate with work functions of 3–5 eV as a function of film thickness. (**d**) Corresponding charge-density distributions are plotted. Simulation parameters for both ZnPc and C_60_ can be found in the supporting information of Ref. [23].

Figure 1a illustrates the calculated distribution of contact-induced charge density in a zinc phthalocyanine (ZnPc) film with thickness of thirty layers. ZnPc molecules presumably adopt edge-on orientation in the film, which has actually been demonstrated [37,38,39]. It is obvious that holes accumulate right at the substrate interface, and that their amount depends on substrate work function (*W*_sub_). When a substrate with higher *W*_sub_ is employed, more holes are generated. Hole concentration is negligible for a *W*_sub_ value of below 5 eV. These findings are explained as follows. If the *E*_F_ of a bottom electrode is located deeper than the HOMO of a ZnPc film, electrons in the ZnPc HOMO are emptied and transferred to the electrode. Since the ionization energy of an edge-on ZnPc molecule in solid state is 4.8 eV [40], the substrate with a *W*_sub_ higher than this value removes electrons from the contacting molecules. 

The hole accumulation leads to a downward potential shift near the electrode interface. Figure 1b shows the evolution of work function in the ZnPc film. Work function gradually decreases as a function of thickness for *W*_sub_ of ≥5 eV, because of the positive charges in the film. The final work function of the ZnPc layer reaches a common value, 4.5 eV. This behavior is known as Fermi-level pinning [41]. The same phenomenon is also referred to as quasi-Fermi level pinning [42]. No significant change of work function, on the other hand, is present when *W*_sub_ is well below the ionization energy of an edge-on ZnPc molecule. The vacuum level aligns throughout the film in this case.

Similar simulations were also carried out for C_60_. Figure 1c illustrates the evolution of charge density in a C_60_ film of thirty layers as a function of thickness. Negative charges are generated at the interface of electrodes with a *W*_sub_ of up to 4 eV, whereas no significant amounts of charged molecules appear when the C_60_ film is formed on the substrate with a *W*_sub_ of over 4 eV. The lower work function a substrate has, the more electrons are accumulated at the electrode interface. Since the LUMO of the C_60_ molecule is 3.98 eV [43], the electrode with a *W*_sub_ lower than this value occupies electrons in the unoccupied states of C_60_.

**Figure 2:**
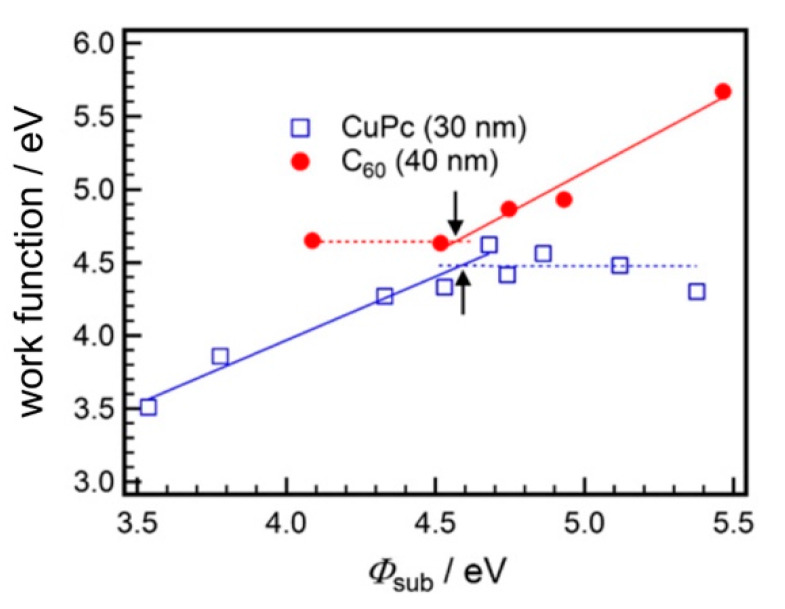
Evolutions of work functions for copper phthalocyanine (CuPc) and C_60_ films. Reprinted with permission [44].

The electron occupation in a C_60_ film alters potential distribution. Figure 1d illustrates the evolution of the work function. For substrates with *W*_sub_ values of 4.5 eV and higher, the work function is constant in a whole range of thicknesses, that is, vacuum level aligns throughout the C_60_ film. On the other hand, an upward shift of the work function is simulated for *W*_sub_ values of 4 eV and below. The final work function of the C_60_ film reaches a common value. Here, the Fermi-level pinning occurs at the interface with low work-function electrodes. The pinning behaviors simulated for metallophthalocyanine and C_60_ films were actually demonstrated by Kelvin probe measurements (Figure 2) [44]. The arrows indicated in Figure 2 show the onset points where the Fermi-level pinning begins.

Recent studies suggest that photocarriers recombine with dark charges near the electrodes, which reduces the fill factor in highly efficient OPVs [45,46]. The occurrence of Fermi-level pinning at the electrode interfaces has been thought to be a must for ohmic contact, but that design is too simple for designing OPV. *W*_sub_ has to be optimized to trade off ohmic contact and the recombination of photocarriers via dark charges.

## 3. Distributions of Potential and Charge Density in Organic Heterojunctions

### 3.1. Fermi-level Pinning at Organic Heterointerfaces

**Figure 3:**
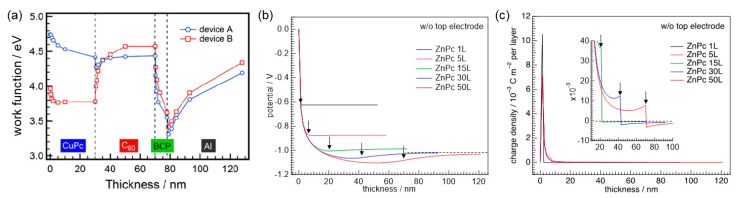
(**a**) Evolutions of measured work functions against thicknesses of components in planar-heterojunction CuPc/C_60_ OPVs. Device A was constructed on a UV-ozone treated ITO substrate, whereas device B was constructed on an ITO covered with an Al film of 1 nm. The latter substrate has a lower work function than a UV-ozone treated ITO, causing a significant change in the evolution of the vacuum level in the C_60_ layer. Reprinted with permission [44]. (**b**) Potential distributions in ZnPc/C_60_ heterojunctions with various thicknesses of ZnPc. The work function of the bottom substrate is assumed to be 5.5 eV. C_60_ thickness was fixed to be fifty layers. The dashed line indicates a saturated value of work function for the C_60_ top layer. (**c**) Depth-resolved charge density profile for the heterojunction with various thicknesses of ZnPc layers. The arrows in (**b**) and (**c**) indicate the positions of each heterointerface. The results shown in (**b**) and (**c**) ignore the impact of top electrodes on the distributions.

We next address energy-level alignment at organic heterointerfaces. One of known phenomena of energetics in organic heterojunctions is that the lineup of the frontier orbitals depends on the *E*_F_ of the bottom layer [15,19,20]. Figure 3a shows an example of a substrate-dependent energy-level alignment. The change in the work function of the C_60_ layer, on top of the CuPc film, became larger when the bottom layer was formed on a substrate with an original work function of <4 eV [44]. The work function of the CuPc layer on this substrate reached 3.8 eV, whereas the CuPc film on a UV-ozone treated indium tin oxide (ITO) had a work function of ~4.5 eV. Since the former value is less than the electron affinity of C_60_, electrons occupy the LUMO of C_60_ upon interface formation, according to the Fermi-Dirac statistic. As a result, an upward shift of 0.8 eV was induced in the C_60_ layer. Note that the final work function of the C_60_ layer reached ~4.5 eV. This value is in good agreement with the onset where the *E*_F_-pinning to the LUMO starts (see Figure 2). The experimental result demonstrated that Fermi-level pinning occurs at organic heterointerfaces.

Pinning behavior was also simulated for ZnPc/C_60_ interfaces with various thicknesses of the bottom phthalocyanine layer (Figure 3b). To calculate the distributions of potential and charge density, *W*_sub_ was set to be 5.5 eV. A common feature in Figure 3b is the downward shift in the ZnPc bottom layer. Since *W*_sub_ exceeds the ionization energy of ZnPc, the positive charges are generated by emptying electrons from the ZnPc HOMO (Figure 3c). Potential distribution near the heterointerface, however, varies with ZnPc thickness. Upward potential shifts are seen across the ZnPc/C_60_ interface for the thickness of fifteen layers and above, whereas no significant shift is present for thinner ZnPc layers. The potential is saturated for the heterointerfaces with ZnPc thicknesses of thirty layers and above, as shown by the horizontal dashed line in Figure 3b. That is, the work function of the outermost C_60_ layer reaches a common value, which is again attributed to Fermi-level pinning at the heterointerface.

**Figure 4:**
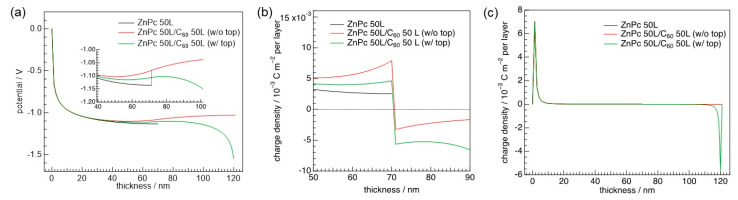
(**a**) Potential distributions of a ZnPc neat film (black) and ZnPc/C_60_ heterojunctions with (green) and without a top electrode (red, work function = 3.5 eV). *W*_sub_ was fixed to be 5.5 eV. (**b**) Charge density profiles near the heterointerface are illustrated. The position of the heterointerface is located at a thickness of 70 nm. The horizontal dashed line indicates a charge density of zero. (**c**) Charge density distributions in a whole range of thicknesses are illustrated.

The simulations revealed that work function of the ZnPc film reduced with increasing ZnPc thickness up to fifty layers from the monolayer, which was indeed demonstrated by KP measurements [47]. The charge density profiles at the heterointerface vary with ZnPc thickness, as shown in Figure 3c. In particular, the electron density in the C_60_ side increases when ZnPc thickness increases. The electron accumulation originates from the Fermi-level pinning of the C_60_ LUMO for the thicker ZnPc layer. As a result, the potential profile, opposite in sign to the depletion layer in the *p*-*n* junction, is realized as seen in Figure 4a.

Note that the rise of potential in the C_60_ layer moves the energy levels of the bottom ZnPc layer toward *E*_F_. This effect further emptied the HOMO of ZnPc right at the heterointerface. As a result, the hole density in the ZnPc side of the heterointerface increases after contact with C_60_ (Figure 4b).

### 3.2. Consideration of Top Electrodes

Distributions of potential and charge density are modified by taking a top electrode into account in the numerical calculations. The green curve in Figure 4a shows the potential shift in an anode (*W*_sub_ = 5.5 eV)/ZnPc/C_60_/cathode (work function = 3.5 eV) structure. It can be seen that the rise of potential across the ZnPc/C_60_ interface decreases as compared to the heterojunction without the top electrode (red curve in Figure 4a). The change in the charge density profile near the heterointerface alters potential distribution (Figure 4b). Placing the electrode on top of the heterojunction decreases (increases) hole (electron) density in comparison to a case without the low-work-function electrode on top. Since thermodynamic equilibrium is achieved throughout the heterojunction, electrons flow from the top to bottom electrodes. The resulting potential gradient lowers the energy levels of both ZnPc and C_60_. This de-pins the HOMO of ZnPc to *E*_F_, whereas the *E*_F_ pinning of the C_60_ LUMO becomes more enhanced. This shift in the energy levels leads to the abovementioned change in charge density profile at the heterointerface with the contact of the top electrode.

### 3.3. Structural Disorder at Donor/Acceptor Interface

**Figure 5:**
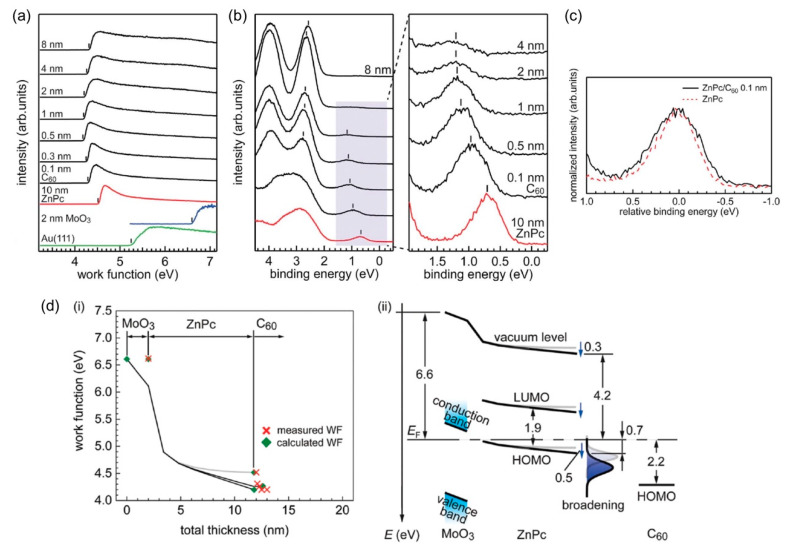
Evolutions of UPS spectra for the ZnPc/C_60_ interface in (**a**) SECO and (**b**) valence regions shown as a function of C_60_ thickness. The right panel in (**b**) expands the valence spectra near the Fermi level. (**c**) Comparison of the HOMO peak for ZnPc before and after deposition of 0.1 nm-thick C_60_. (**d**) (i) The shift of the work function. Black lines indicate electrostatic potentials. The measured and simulated work functions are denoted by red crosses and green diamonds, respectively. (ii) The energy diagram for the heterostructure is illustrated. The broadening of the ZnPc HOMO produces positive charges and then a downward energy shift is induced. Reprinted with permission [21].

The electrostatic model used for the simulations assumes layer-by-layer growth of organic films and the formation of a sharp interface. However, realistic organic heterointerfaces involve molecular reorientation [40], chemical interaction [48], and the formation of an amorphous mixed phase [49]. As for phthalocyanine/C_60_ interfaces, the structural disordering upon the formation of the interface was speculated based on the analysis of the valence electronic structure. Figure 5a,b show the UPS spectra for the ZnPc (10 nm)/C_60_ interface prepared on MoO_3_-covered Au(111) in the secondary cutoff (SECO) and valence regions, respectively. The simulation for this heterojunction predicted vacuum-level alignment at the heterointerface [21]. The SECO spectra, however, demonstrated downward and then upward shifts of the vacuum level (Figure 5a). Concurrently, the peak of the ZnPc HOMO shifts toward higher binding energy, whereas the C_60_ HOMO peak shifts toward lower binding energy. This result clearly suggests that the formation of interface dipoles does not rationalize the opposite shift of the ZnPc HOMO to C_60_ HOMO. As shown in Figure 5c, we noted that the HOMO of ZnPc became broader upon the deposition of C_60_ [21]. This broadening can indicate an increase in structural inhomogeneity, because the energy distributions of the HOMO and LUMO rely on the distribution of polarization energy in the system. Actually, a time-of-flight secondary ion mass spectroscopy (TOF-SIMS) study suggested intermixing at the edge-on CuPc/C_60_ interface [50], which probably leads to an increase in the exponential DOS in the energy gap [51]. Considering the increased tailing of the HOMO of ZnPc right at the interface, we simulated the potential distribution at the ZnPc/C_60_ interface (Figure 5d). This observation was reproduced by the simulations. The mechanism of energy-level alignment involving the increase in structural inhomogeneity was understood as follows. Tailing states of ZnPc reach the Fermi level upon the contact with C_60_. Fermi-Dirac statistics require the emptying of the electrons from the small occupied DOS within an energy gap. As a result, positive potential is generated, shifting the energy levels downward. Concurrently, ZnPc work function decreases. *E*_F_ is then pinned to the C_60_ LUMO, which leads to an upward shift in the thicker C_60_. The findings in this study suggest that an understanding of molecular arrangement at the heterointerface is essential. In particular, occurrence of intermixing and structural disordering for arbitrary heterointerfaces should thus be investigated thoroughly. STM studies have been reported for molecular bilayers to address this issue [52,53,54], but investigating the heterojunction with thicknesses relevant to realistic devices will be necessary.

### 3.4. Energy-Level Alignment in Reversed Stacking

In a planar-heterojunction OPV cell, donor molecules make contact solely with an anode and acceptor molecules make contact with a cathode. Molecular composition near the electrodes in a bulk heterojunction, however, becomes complicated in general, because both donor and acceptor may interface with each electrode when a donor/acceptor blend is formed by co-evaporation or spin-coating. Thus, a reversed stacking, for instance, an anode/acceptor/donor junction, would exist in a bulk-heterojunction OPV cell. This section addresses energy-level alignment in this uncommon heterostructure. 

**Figure 6:**
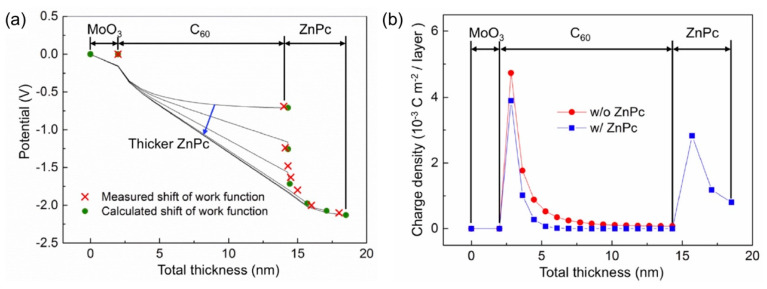
(**a**) Evolutions of measured (red crosses) and simulated (green circles) work functions. Black lines indicate electrostatic potential distributions within the respective layers. (**b**) Charge density distributions before (red) and after (blue) completion of the heterostructure. Reprinted with permission [23].

The heterojunction that we investigated consisted of a MoO_3_-covered Au(111)/C_60_ 12 nm/ZnPc structure. Analysis of UPS spectra clarified that the work function decreased upon depositing the C_60_ film onto the substrate (Figure 6a). The simulations showed that positive charges are present in the C_60_ layer near the interface with MoO_3_ through the emptying electrons from the HOMO of C_60_ by the high-work-function metal oxide (Figure 6b). The hole accumulation then leads to a downward shift of the vacuum level. Completion of the heterostructure by depositing ZnPc on top of the C_60_ film led to a huge vacuum-level shift. As seen in Figure 6a, work function decreases by ~1.5 eV upon completing the heterointerface. The simulations demonstrated that a large fraction of the energy shift was attributed to the change in potential in the C_60_ layer [23]. The rest of the decrease in work function originates from band-bending-like shifts in the ZnPc top layer. Note that the potential profile demonstrated is totally different from that for the standard stacking sequence, anode/ZnPc/C_60_ [21]. Therefore, symmetric energy-level alignment does not always hold, as has already been pointed out [19]. 

Analysis of the charge density profiles, shown in Figure 6b, helped form an understanding of the mechanism of energy-level alignment in this heterostructure. The work function of the C_60_ bottom layer was measured to be 6.0 eV, which is still high enough compared to the ionization energy of the top ZnPc layer. Thus, upon the deposition of ZnPc onto the C_60_ film, the electron of the ZnPc HOMO gets emptied and then positive charges are generated in the ZnPc side at the interface (Figure 6b). It is worth mentioning that electrons in the C_60_ side at the heterointerface were absent in the simulation results. This indicates that interface dipoles are not formed in spite of the presence of positive charges on the ZnPc side. Since the charges in the ZnPc layer induce electrostatic fields across the C_60_/ZnPc heterojunction, a linear potential gradient appears in the C_60_ layer (black lines in Figure 6a). The lowering of the potential reduces the hole density near the MoO_3_ interface upon the formation of the heterointerface, as shown by the blue curve in Figure 6b. Since the potential gradient moves the HOMO away from *E*_F_, the pinning of the C_60_ HOMO to *E*_F_ decreases. As a result, the concentration of holes in the C_60_ layer decreases after completing the heterostructure.

## 4. Conclusions

In summary, contact-induced charges and resulting electrostatic fields govern the energetics at electrode/organic and organic heterointerfaces at thermodynamic equilibrium. Our studies on organic interfaces, related to conventional OPV cells, showed that substrate work-function and layer thicknesses are critical for determining the work function of organic films. Variation of the work function influences the magnitude of built-in potential at electrode/organic and organic heterointerfaces. Due to the Fermi-level pinning, a large fraction of dark charge accumulates at electrode interfaces, which may cause photocarrier recombination, as reported in Refs. [45,46]. It is however remarkable that contact-induced charges are present also at the donor/acceptor interface. The interface dark charges may assist exciton separation based on a simple electrostatic calculations [55], although experimental validation is necessary. We found that the top electrode and structural disorder considerably influence the charge distribution. The latter factor may be present also in bulk heterojunction, as a systematic study via cyclic voltammetry and UPS suggests the shortening of polymer conjugation length in an amorphous mixed phase with fullerene [49]. The DOS of the constituents in bulk heterojunction is not accessible by surface-sensitive UPS, because the polymer cap layer on the blend surface, segregated due to its low surface energy, can screen the buried molecular composition [56]. If the shape of DOS is modified in bulk heterojunction due to structural disordering (broadening and/or tailing), energy-level alignment near the electrode should be altered. This could be probed by measuring thickness-dependent work function with UPS or KP. We, however, point out that blending two dissimilar molecules alters ionization energy and electron affinity, due to the quadrupolar electrostatic field [57,58]. Taking this effect into account under assumption of identical molecular composition over a whole thickness range, fitting the thickness-dependent evolution of work function with electrostatic simulation will reveal information regarding static disorder in the bulk heterojunction for donor and acceptor. However, this analysis would be challenging.

Finally, let us add some comments on how to address state-of-the-art OPVs from the viewpoint of the energetic landscape. First, as mentioned just above, implementing the influence of molecular quadrupolar fields [58,59] on changes in ionization energy and electron affinity at the donor/acceptor interface will give us a more precise energetic landscape at the interface. Second, based on the findings for conventional devices, the character of electronic structures of organic semiconductors already in use has to be considered. Efficient OPVs that have recently been reported on constitute donors with higher ionization energy than ZnPc and acceptors with a similar ionization energy or electron affinity to the donor [60,61,62]. These differences in electronic properties between conventional and recent donor/acceptor pairs must differentiate the energy-level lineup in OPVs. Electrostatic simulations for state-of-the-art donor/acceptor pairs are ongoing and will be reported elsewhere.
